# Fish Oil Ameliorates Deoxynivalenol-Induced Liver Injury Through Modulating Ferroptosis Signaling Pathway in Weaned Pigs

**DOI:** 10.3390/ani16081234

**Published:** 2026-04-17

**Authors:** Jiasi Liu, Minfang Zhang, Mohan Zhou, Junjie Guo, Shaokui Chen, Kan Xiao, Yulan Liu

**Affiliations:** Hubei Key Laboratory of Animal Nutrition and Feed Science, Wuhan Polytechnic University, Wuhan 430023, China; 15897721405@163.com (J.L.); asx12zxq4@163.com (M.Z.); chocome2@126.com (M.Z.); dkxyyjs123@163.com (J.G.); loveskchen@163.com (S.C.)

**Keywords:** deoxynivalenol, fish oil, liver injury, piglets, ferroptosis

## Abstract

Fish oil (FO) has been shown to confer beneficial effects on hepatic diseases in both humans and animals. This study aimed to investigate whether dietary fish oil (FO) supplementation alleviates deoxynivalenol (DON)-induced liver injury by modulating the ferroptosis signaling pathway in weaned piglets. Twenty-four weaned piglets were allocated to a 2 × 2 factorial design, with the main factors consisting of dietary treatment (5% corn oil or 5% FO supplementation) and DON exposure (basal diet or diet contaminated with 4 mg/kg DON). After 21 days of dietary treatment, piglets were euthanized for collection of blood and liver samples. Dietary FO significantly attenuated DON-induced hepatic structural damage and inflammatory infiltration. Specifically, FO supplementation reduced the activities of aspartate transaminase (AST) and alkaline phosphatase (ALP), as well as the AST/alanine aminotransferase (ALT) ratio following DON exposure. Dietary FO also decreased malondialdehyde (MDA) concentrations in both the liver and serum, lowered hepatic 4-hydroxynonenal (4-HNE) level and Fe^2+^ content, and increased hepatic glutathione (GSH) content. Moreover, dietary FO ameliorated ultrastructural liver damage induced by DON. Furthermore, DON significantly downregulated the mRNA levels of multiple genes associated with iron metabolism and ferroptosis, including heat shock protein beta-1 (HSPB1), acyl-CoA synthetase long chain family member 4 (ACSL4), and arachidonate 15-lipoxygenase (ALOX15), and upregulated the mRNA levels of transferrin (TF), ferritin heavy chain (FTH), solute carrier family 7 member 11 (SLC7A11), and transferrin receptor 1 (TFR1). Dietary FO counteracted these alterations by decreasing the mRNA of SLC7A11, TFR1, FTH, and TF after DON exposure. Finally, FO significantly decreased the protein expression of SLC7A11, iron-responsive element-binding protein 2 (IREB2), and FHT1 and increased the GPX4 protein expression following DON exposure. These findings suggest that FO may ameliorate DON-induced liver injury in weaned piglets, possibly through suppressing the ferroptosis signaling pathway.

## Simple Summary

This study investigated the potential hepatoprotective effects of fish oil in liver injury induced by deoxynivalenol (DON) in weaned piglets. The research focused on the role of ferroptosis, an iron-dependent form of regulated cell death characterized by excessive lipid peroxidation, which is implicated in liver damage. Our findings demonstrate that dietary fish oil may effectively attenuate DON-induced hepatic injury. The protective effects appear to be associated with reduction in serum biomarkers of hepatic damage. Furthermore, fish oil mitigated oxidative stress by decreasing concentrations of lipid peroxidation products and enhancing hepatic antioxidant capacity. Crucially, fish oil counteracted the DON-induced dysregulation of key genes and proteins of the ferroptosis pathway, potentially to the restoration of iron metabolism and glutathione-dependent antioxidant defense. In conclusion, dietary fish oil may confer protection against DON-induced liver injury in weaned piglets, which is likely mediated by suppressing the ferroptosis signaling pathway.

## 1. Introduction

Deoxynivalenol (DON), a mycotoxin primarily produced by *Fusarium graminearum* and *F. culmorum* [1], is one of the most prevalent contaminants in cereals and their by-products [2,3]. Among livestock, piglets are the most sensitive species to DON exposure, which can induce acute symptoms such as diarrhea, anorexia, and vomiting, as well as chronic effects including growth retardation and immune dysfunction [4,5]. Studies have demonstrated that DON exposure can induce multi-organ damage in piglets, particularly impairing the intestine, liver, and kidneys [6,7]. As the primary detoxification organ, the liver plays a critical role in mitigating mycotoxin toxicity. In vivo experiments have revealed that DON disrupts hepatic architecture, triggers inflammatory responses, and promotes hepatocyte apoptosis [8]. Mechanistically, DON elevates reactive oxygen species (ROS) levels while suppressing antioxidant enzyme activity, thereby exacerbating hepatic oxidative stress and ultimately contributing to liver injury and functional impairment [9]. Given these detrimental effects, developing effective nutritional interventions to alleviate DON-induced liver damage in piglets is of critical importance.

Fish oil (FO), rich in long-chain omega-3 polyunsaturated fatty acids (n-3 PUFAs) such as eicosapentaenoic acid (EPA) and docosahexaenoic acid (DHA), has demonstrated beneficial effects on various human and animal diseases [10]. Previous studies have shown that FO can regulate hepatic lipid metabolism while mitigating oxidative stress and inflammation [11,12]. However, its potential to alleviate liver injury induced by DON exposure in porcine models remains to be elucidated.

Ferroptosis is a newly identified iron-dependent programmed cell death characterized by excessive lipid peroxidation and dysregulation of amino acid antioxidant systems. Ultrastructural hallmarks of ferroptosis include mitochondrial degeneration, manifested by reduced or absent cristae and ruptured outer membranes [13]. Ferroptosis is tightly regulated by three key biological processes: iron metabolism, oxidative stress, and cytotoxic amino acid metabolism. Iron homeostasis plays a pivotal role in ferroptosis initiation. Concurrently, disruption of the amino acid antioxidant system, particularly glutathione (GSH) depletion, critically contributes to ferroptosis. GSH, synthesized from glutamate, cysteine, and glycine, serves as a major cellular redox buffer. Under stress conditions, inhibition of system Xc- impairs cystine uptake, diminishing GSH synthesis and subsequently reducing GPX4 activity, a key enzyme responsible for lipid peroxide detoxification. This cascade leads to ROS accumulation and lethal lipid peroxidation, driving ferroptosis execution. Notably, lipid peroxidation represents the biochemical cornerstone of ferroptosis. The process is amplified by the upregulation of key lipid-metabolizing enzymes, including Acyl-CoA synthetase long-chain family member 4 (ACSL4), lysophosphatidylcholine acyltransferase 3 (LPCAT3), and arachidonate 15-lipoxygenase (ALOX15). These enzymes enhance the incorporation of polyunsaturated fatty acids (PUFAs) into membrane phospholipids, rendering cells more susceptible to oxidative damage. Emerging evidence further links ferroptosis and lipid peroxidation to the pathogenesis of liver diseases, highlighting its potential therapeutic relevance [13,14].

Therefore, the purpose of this study was to explore whether FO could alleviate liver injury after DON exposure through modulating the ferroptosis signaling pathway in weaned piglets. We hypothesized that FO supplementation may alleviate DON-induced liver injury in weaned piglets by regulating the ferroptosis signaling pathway, thereby improving the liver’s antioxidant capacity and reducing lipid peroxidation through the modulation of ferroptosis-related genes and proteins.

## 2. Materials and Methods

### 2.1. Experimental Animals and Design

The animal protocol for this study was approved by the Institutional Animal Care and Use Committee of Wuhan Polytechnic University (WPU201911003). The experiment was randomly arranged as a 2 × 2 factorial design with two factors: dietary oil (5% corn oil or 5% FO) and DON exposure (basal diet vs. DON-contaminated diet). Twenty-four weaned piglets (Large White × Landrace, barrows, 35-days old, initial body weight (BW) 7.5 ± 0.21 kg) were randomly divided into four groups: CO group (5% corn oil), FO group (5% FO), CO + DON group (5% corn oil with DON), and FO + DON group (5% FO with DON). Piglets were housed individually in pens and had free access to feed and water. The methods of DON cultivation followed those by Wang et al. [15]. The concentration of DON in the diet was chosen according to previous research [16,17] and our preliminary study. The FO was purchased from Fujian Gaolong Marine Biotechnology Co., Ltd. (Fuzhou, Fujian, China). The basal diet was formulated to meet or exceed the nutrient requirements of growing pigs, as recommended by NRC (2012) (Table S1). The DON concentrations in the basal diet and DON-contaminated diet were 0.3 mg/kg and 4 mg/kg, respectively. The fatty acid composition of 5% corn oil and 5% FO is shown in Table S2. The pigs were adapted for 7 d. After 21 d, piglets were weighed and blood samples were collected. Then piglets were anesthetized by intramuscular injection with Zoletil^®^ 50 (10 mg/kg BW, Virbac, Carros, France) to euthanize for liver samples.

### 2.2. Blood and Liver Sampling

The procedures for blood and liver sample collection followed Li et al. [18]. Briefly, blood samples were collected from the jugular vein of weaned piglets after 21 days of feeding period, immediately after the piglets were weighed and before anesthesia. Then blood samples were centrifuged at 3500 rpm for 10 min to obtain serum. The serum was then stored at −80 °C for biochemical analysis. A 0.5 × 0.5 × 0.5 cm tissue block was excised from the middle part of the right lateral lobe. A portion of the sampled liver tissue was immediately fixed in 4% paraformaldehyde or 2.5% glutaraldehyde solutions for histological analysis, while the remaining portion was rapidly frozen in liquid nitrogen for subsequent protein and gene expression analysis.

### 2.3. Serum Biochemical Parameters

Serum alanine aminotransferase (ALT), aspartate aminotransferase (AST), and alkaline phosphatase (ALP) levels were measured using a Hitachi 7020 Automatic Biochemical Analyzer according to the kit instructions (AST: KP100, ALT: KP622, ALP: EK872, Fujifilm Wako Pure Chemical Co., Tokyo, Japan).

### 2.4. Liver Morphology

Liver samples were dehydrated, embedded in paraffin, and sectioned at 5 μm and finally stained with hematoxylin-eosin (HE). The liver morphology was observed under an Olympus light microscope at 200× magnification (Olympus Co., Tokyo, Japan). For quantitative assessment of hepatic inflammatory infiltration area ratio, five non-overlapping high-power fields were randomly selected from each HE-stained liver section. The area of inflammatory infiltration was measured using Image J software (National Institutes of Health, Bethesda, MD, USA), and the hepatic inflammatory infiltration area ratio (%) was calculated.

### 2.5. Ultrastructural Observation

Liver samples for electron microscopy were prepared using standard procedures (fixation, dehydration, embedding, sectioning, and staining) as described by Gu et al. [19].

Specifically, after sampling, 0.5 × 0.5 × 0.5 cm liver tissue blocks were immediately fixed in 2.5% glutaraldehyde. Following three rinses with PBS, the samples were post-fixed in 1% osmium tetroxide. Subsequently, the samples were dehydrated using a graded ethanol series, embedded in Epon 812 resin, and sectioned into 60–80 nm ultrathin sections. The sections were stained with 2% uranyl acetate and lead citrate prior to observation under a transmission electron microscope (TEM, Tecnai, FEI, Hillsboro, OR, USA) at 15,000× magnification.

### 2.6. Hepatic and Serum Redox Status Analysis

The concentrations of GSH and malondialdehyde (MDA) in liver and serum were measured using commercial kits (GSH assay kit and MDA assay kit, Nanjing Jiancheng Bioengineering Institute, Nanjing, China).

### 2.7. Hepatic 4-HNE, Fe^2+^ and GPX4 Contents Analysis

The concentration of 4-hydroxynonenal (4-HNE) in liver was determined using a porcine 4-HNE ELISA kit (RuiXin Biotechnology, Quanzhou, China). Fe^2+^ and GPX4 contents were also detected by Fe^2+^ and GPX4 assay kits (Nanjing JianchengBioengineering Ins. Nanjing, China).

### 2.8. mRNA Abundance

The mRNA expression of ferroptosis-related genes, including ferroportin (FPN), transferrin (TF), ferritin heavy chain (FTH), heat shock protein beta-1 (HSPB1), solute carrier family 7 member 11 (SLC7A11), transferrin receptor 1 (TFR1), acyl-CoA synthetase long chain family member 4 (ACSL4), and arachidonic acid 15-lipoxygenase (ALOX15), in liver were measured by real-time PCR. Total RNA was extracted from liver tissue using TRIzol reagent (TaKaRa Biotechnology, Beijing, China) according to the manufacturer’s instructions. The integrity of the extracted RNA was verified by 1.5% agarose gel electrophoresis, which confirmed the presence of clear 28S and 18S ribosomal RNA bands without obvious degradation. The purity and concentration of the RNA were quantitatively determined using a NanoDrop 2000 spectrophotometer (Thermo Fisher Scientific, Waltham, MA, USA). The absorbance ratios at 260 nm/280 nm (OD260/280) and 260 nm/230 nm (OD260/230) were measured to assess RNA purity. The concentration of the RNA was accurately quantified based on the absorbance value at 260 nm. After quantification, RNA was reverse-transcribed into cDNA. The results were analyzed using the 2-ΔΔCt method (Livak and Schmittgen, 2001) [20], with β-actin as the housekeeping gene. Relative mRNA abundance for each target gene was normalized to the corn oil (CO) group. The primer sequences used are provided in Table S3.

### 2.9. Protein Expression

The expression of ferroptosis-related proteins was evaluated by Western blotting. The following primary antibodies were used: rabbit anti-FTH1 (1:1000, Abcam, Cambridge, UK), rabbit anti-TF (1:1000, ABclonal, Wuhan, China), rabbit anti-IREB2 (1:1000, Proteintech, Wuhan, China), rabbit anti-GPX4 (1:200, Cayman, MI, USA), and mouse anti-β-actin (1:1000, Sigma Chemical Incorporated, Saint Louis, MO, USA). After incubation with primary antibodies, membranes were washed and then incubated with an HRP-conjugated secondary antibody (anti-rabbit IgG, 1:5000, AntGene Biotech, Wuhan, China) at room temperature. Protein bands were visualized using an enhanced chemiluminescence (ECL) kit (Amersham, Piscataway, NJ, USA) and detected with an Alpha Innotech Imaging System (Syngene, Cambridge, UK). Relative protein abundance was calculated as the ratio of target protein density to β-actin protein density.

### 2.10. Statistical Analysis

All data were analyzed using SPSS Statistics 22 (IBM SPSS, Chicago, IL, USA). Results were presented as means ± SE. A two-way analysis of variance (ANOVA) under a general linear model was applied to assess the effects of DON, FO, and their interaction. The statistical model included the main effects of DON (control vs. DON treatment), FO (CO vs. FO), and their interaction. Prior to two-way analysis of variance (ANOVA), the normality of data distribution was assessed using the Shapiro–Wilk test, and the homogeneity of variances was verified using Levene’s test. Multiple comparison tests were performed using Duncan’s multiple comparisons. Six pigs per treatment group were used as independent biological replicates (*n* = 6). Differences were considered significant at *p* < 0.05.

## 3. Results

### 3.1. Effect of Dietary FO on Liver Morphology of Piglets After DON Exposure

**Figure 1:**
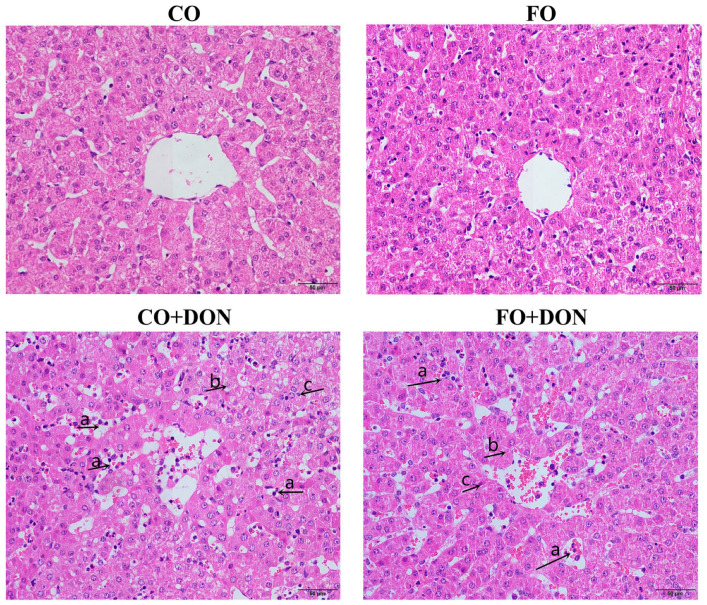
Effect of dietary FO on liver morphology of piglets after DON exposure. Representative photomicrographs of liver (HE-stained) from piglets. CO group: piglets fed 5% corn oil; FO group: piglets fed 5% FO diet; DON group: piglets fed 5% CO diet and 4 mg/kg DON; FO + DON group, piglets fed diet with 5% FO diet and 4 mg/kg DON. Arrows indicate: (a) Inflammatory cell infiltration, (b) Karyolysis, (c) Nuclear pyknosis. The magnification was 200×. CO: corn oil; DON, deoxynivalenol; FO, fish oil.

**Figure 2:**
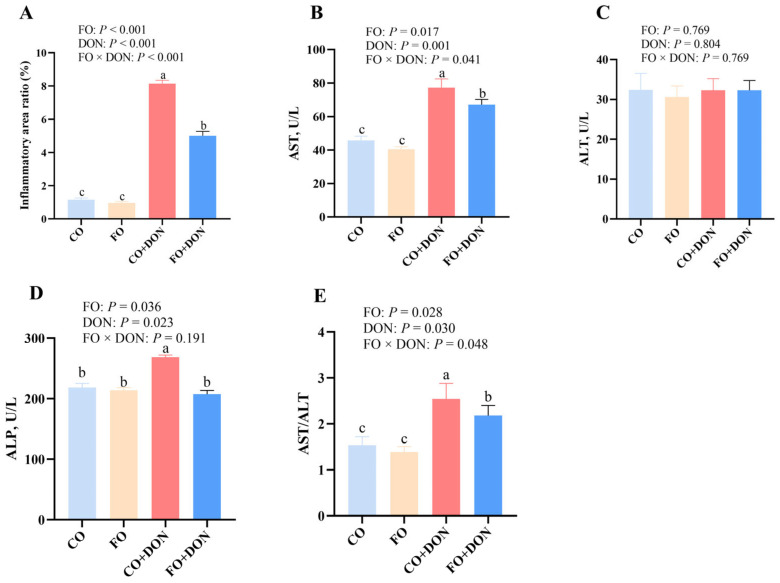
Effect of dietary FO on hepatic inflammatory infiltration area ratio and liver function of piglets after DON exposure. CO group: piglets fed 5% CO; FO group: piglets fed 5% FO diet; DON group: piglets fed 5% CO diet and 4 mg/kg DON; FO + DON group, piglets fed diet with 5% FO diet and 4 mg/kg DON. (**A**) hepatic inflammatory infiltration area ratio. (**B**) AST activity. (**C**) ALT activity. (**D**) ALP activity. (**E**) AST/ALT. Values are means ± SE, *n* = 6. ^a–c^ Different letters represent a significant difference. Differences were considered significant for values of *p* ≤ 0.05. CO: corn oil; DON, deoxynivalenol; FO, fish oil; ALP: alkaline phosphatase; ALT: alanine aminotransferase; AST: aspartate transaminase.

To investigate the effects of FO on hepatic structure after DON exposure, we examined the liver histology of piglets. No obvious morphologic changes were observed in pigs fed corn oil (CO) or fish oil (FO) diets without DON (Figure 1). After DON exposure, morphologic changes were observed, including inflammatory cell infiltration, karyolysis, and nuclear pyknosis. Furthermore, quantitative analysis showed that DON exposure significantly increased the hepatic inflammatory infiltration area ratio, while dietary FO significantly reduced inflammatory cell infiltration after DON exposure (*p* < 0.05) (Figure 2A). Compared with DON-challenged pigs fed CO, those fed FO showed significantly less hepatic structural injury.

### 3.2. Effect of Dietary FO on Liver Function of Piglets After DON Exposure

To investigate the effects of FO on hepatic function after DON exposure, we measured AST, ALT, and ALP activities in serum. FO significantly decreased the activities of AST and ALP, as well as the AST/ALT ratio (*p* < 0.05) after DON exposure, but had no significant effect on ALT activity (Figure 2B–E).

### 3.3. Effect of Dietary FO on Liver Ultrastructure of Piglets After DON Exposure

**Figure 3:**
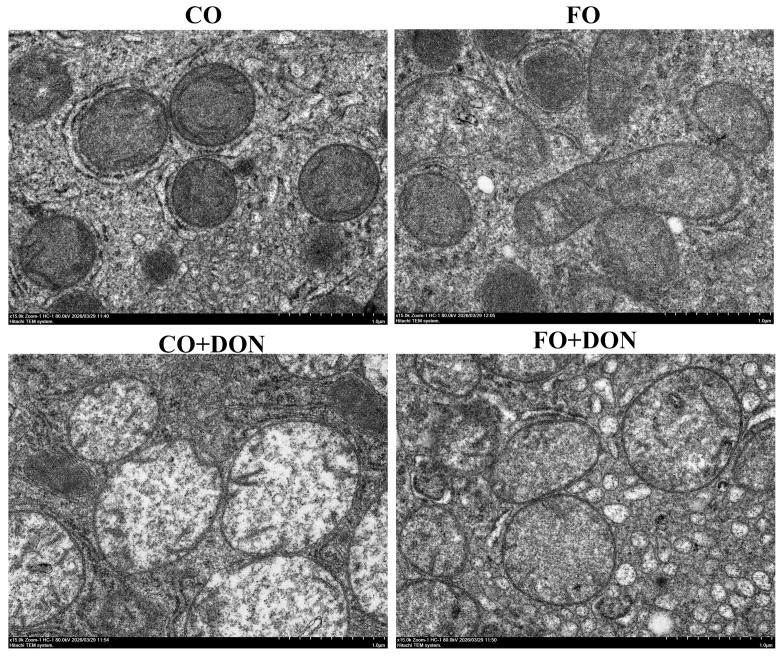
Effect of dietary FO on liver ultrastructure of piglets after DON exposure. Representative ultrastructure photomicrographs of liver (TEM) from piglets. CO group: piglets fed 5% CO; FO group: piglets fed 5% FO diet; DON group: piglets fed 5% CO diet and 4 mg/kg DON; FO + DON group, piglets fed diet with 5% FO diet and 4 mg/kg DON. The magnification was 15,000×. CO: corn oil; DON, deoxynivalenol; FO, fish oil; TEM: TEM, transmission electron microscope.

To examine the effects of FO on hepatic ultrastructure after DON exposure, we observed liver ultrastructure. DON-challenged piglets exhibited ultrastructure damage including mitochondria vacuolation, and reduced cristae, compared to piglets fed CO. However, dietary FO alleviated the DON-induced ultrastructural alterations (Figure 3).

### 3.4. Effect of Dietary FO on Hepatic and Serum Redox Status of Piglets After DON Exposure

**Figure 4:**
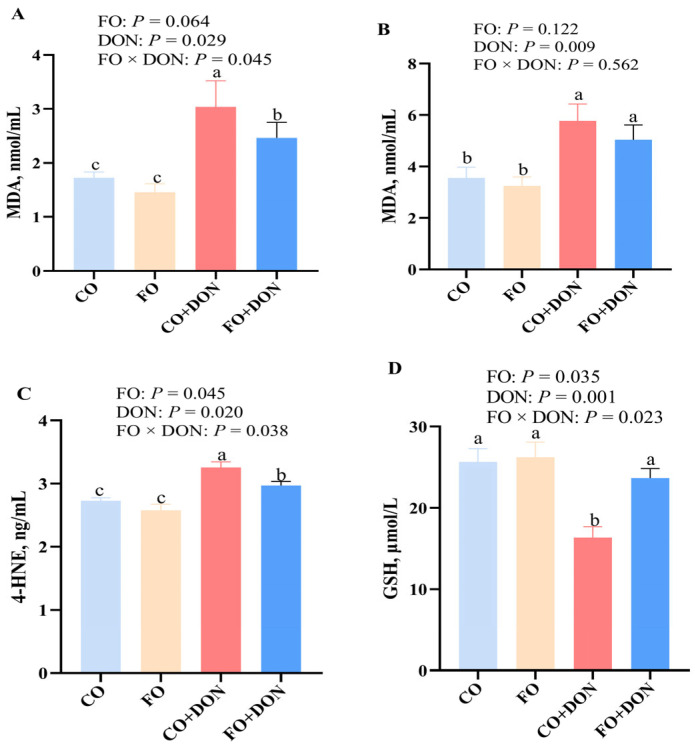
Effect of dietary FO on hepatic and serum redox status of piglets after DON exposure. CO group: piglets fed 5% CO; FO group: piglets fed 5% FO diet; DON group: piglets fed 5% CO diet and 4 mg/kg DON; FO + DON group, piglets fed diet with 5% FO diet and 4 mg/kg DON. (**A**) MDA in serum. (**B**) MDA in liver. (**C**) 4-HNE in liver. (**D**) GSH in liver. Values are means ± SE, *n* = 6. ^a–c^ different letters represent a significant difference. Differences were considered significant for values of *p* ≤ 0.05. 4-HNE: 4-hydroxynonenal; CO: corn oil; DON, deoxynivalenol; FO, fish oil; GSH: glutathione; MDA: malondialdehyde.

To assess the effect of FO on redox status in piglets, we measured GSH and MDA levels in the liver and serum. DON exposure significantly decreased hepatic GSH levels (*p* < 0.05), while FO supplementation increased GSH levels (Figure 4). There was a significant interaction between DON and FO on hepatic GSH (*p* < 0.05). DON exposure significantly increased (*p* < 0.05) MDA concentration in both liver and serum. In contrast, FO supplementation decreased MDA levels (*p* < 0.05) in the liver and serum. No DON × FO interaction was observed for hepatic MDA levels.

### 3.5. Effect of Dietary FO on 4-HNE Concentration in Liver After DON Exposure

To further evaluate the effect of FO on hepatic oxidative stress in piglets, we measured 4-HNE levels in liver. DON exposure increased hepatic 4-HNE levels (*p* < 0.05). Dietary FO decreased 4-HNE levels compared with the CO group. A FO × DON interaction was observed for the 4-HNE (*p* < 0.05) level in which FO decreased 4-HNE levels in piglets fed with DON (Figure 4C), whereas 4-HNE did not differ in non-DON-treated piglets.

### 3.6. Effects of Dietary FO on mRNA Abundance of Hepatic Ferroptosis Markers After DON Exposure

**Figure 5:**
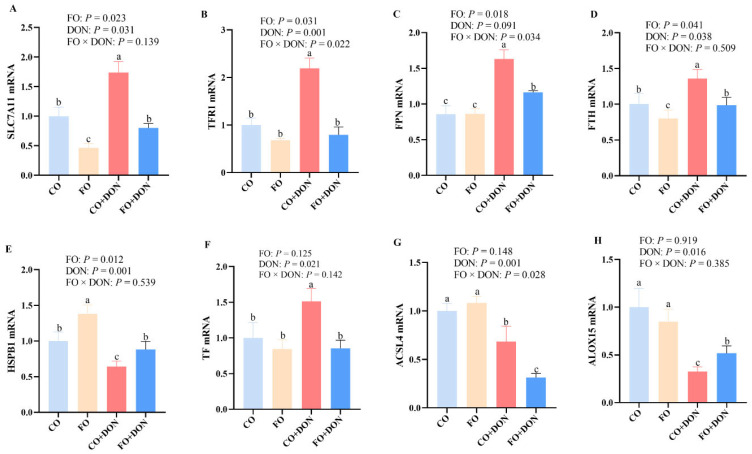
Effects of dietary FO on mRNA abundance of hepatic ferroptosis markers after DON exposure. CO group: piglets fed 5% corn oil; FO group: piglets fed 5% fish oil diet; DON group: piglets fed 5% corn oil diet and 4 mg/kg DON; FO + DON group, piglets fed diet with 5% fish oil diet and 4 mg/kg DON. (**A**) SLC7A11 mRNA. (**B**) TFR1 mRNA. (**C**) FPN mRNA (**D**) FTH mRNA. (**E**) HSPB1 mRNA. (**F**) TF mRNA. (**G**) ASCL4 mRNA. (**H**) ALOX15 mRNA. (**A**) MDA in serum. (**D**) GSH in liver.Values are means ± SE, *n* = 6. ^a–c^ Means without a common letter differ (*p* < 0.05). ACSL4: acyl-CoA synthetase long chain family member 4; ALOX15: arachidonate 15-lipoxygenase; CO: corn oil; DON, deoxynivalenol; FO, fish oil; FPN: ferroportin; FTH: ferritin heavy chain; HSPB1: heat shock protein beta-1; SLC7A11: solute carrier family 7 member 11; TF: transferrin; TFR1: transferrin receptor 1.

To explore the effects of FO on ferroptosis-related genes after DON exposure, we measured the expression of ferroptosis-related genes in liver. DON significantly downregulated the mRNA levels of multiple genes associated with iron metabolism and ferroptosis, including HSPB1, ACSL4, and ALOX15, and upregulated the mRNA levels of TF, FTH, SLC7A11, and TFR1. Significant FO × DON interactions were observed for TFR1 and ACSL4 mRNA levels, in which FO supplementation decreased the mRNA expression of TFR1 and ACSL4 in DON-treated piglets; however, TFR1 and ACSL4 mRNA expression did not differ in non-DON treated piglets (Figure 5).

### 3.7. Effects of Dietary FO on Protein Expression of Hepatic Ferroptosis Markers After DON Exposure

**Figure 6:**
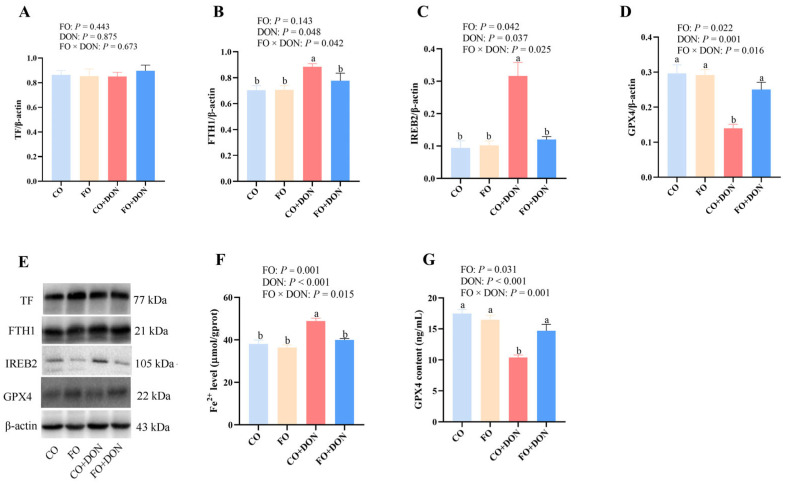
Effects of dietary FO on protein expression of hepatic ferroptosis markers after DON exposure. CO group: piglets fed 5% CO; FO group: piglets fed 5% FO diet; DON group: piglets fed 5% CO diet and 4 mg/kg DON; FO + DON group, piglets fed diet with 5% FO diet and 4 mg/kg DON. (**A**–**D**) Ferroptosis protein expression. (**E**) Representative bands. (**F**) Fe^2+^ content. (**G**) GPX content. Values are means ± SE, *n* = 6. ^a,b^ Different letters represent a significant difference. Differences were considered significant for values of *p* ≤ 0.05. CO: corn oil; DON, deoxynivalenol; FO, fish oil; FTH1: ferritin heavy chain 1; GPX4: glutathione peroxidase 4; IREB2: iron-responsive element-binding protein 2; TF: transferrin.

To determine whether FO influences ferroptosis-related proteins, we examined the expression of ferroptosis-related proteins in liver. DON exposure significantly decreased the protein expression of GPX4 and increased the protein expression of FTH1 and IREB2 (*p* < 0.05). FO supplementation, in turn, significantly reduced the protein expression of FTH1 and IREB2 (*p* < 0.05), and increased the protein expression of GPX4 (*p* < 0.05). Significant FO × DON interactions were observed for FTH1 and IREB2 protein expression, in which FO supplementation decreased the protein expression of FTH1 and IREB2 in DON-treated piglets; however, these proteins did not differ in non-DON-treated piglets (Figure 6A–E). A significant interaction was observed for GPX4 protein expression, in which FO supplementation increased the protein expression of GPX4 in DON-treated piglets; however, GPX4 protein expression did not differ in non-DON-treated piglets.

Moreover, DON exposure significantly increased the contents of Fe^2+^ content (*p* < 0.05) and decreased GPX4 activity (*p* < 0.05) in liver. FO supplementation, in turn, significantly reduced the content of Fe^2+^ and increased the GPX4 activity (*p* < 0.05). Significant FO × DON interactions were observed for Fe^2+^ content and GPX4 activity, in which FO supplementation increased the GPX4 activity and decreased Fe^2+^ content in DON-treated piglets; however, these indices did not differ in non-DON-treated piglets (Figure 6F,G).

## 4. Discussion

An intact hepatic structure is essential for normal function of liver. In this experiment, we observed that DON exposure induced liver injury, while FO supplementation alleviated both structural and functional damage caused by DON. Quantitative histopathological analysis further confirmed that DON significantly increased the hepatic inflammatory infiltration area ratio, whereas FO effectively reduced inflammatory cell infiltration, indicating that FO mitigated hepatic inflammatory damage induced by DON. Consistent with our results, Chen et al. [21] reported that FO helped preserve hepatic architecture and function in weaned pigs challenged with lipopolysaccharide (LPS). Similarly, Zhang et al. [22] demonstrated that FO protected liver morphology and attenuated liver inflammation following LPS exposure in piglets. ALT and AST are widely recognized as sensitive biomarkers of hepatic damage [18], while ALP activity in serum is also an important indicator of liver function [23]. In the present study, DON increased the AST, ALP, and AST/ALT in serum, while FO decreased the AST, ALP, and AST/ALT after DON exposure, suggesting a protective effect of FO on liver function. In support of this, Wu et al. [24] found that prolonged dietary exposure to 3 mg/kg DON significantly increased serum AST and ALP activities in 60-day-old growing pigs.

Liver injury is closely related to cell death. Ferroptosis, a recently identified form of metabolically regulated programmed cell death, differs from apoptosis, autophagy, and necrosis in both characteristics and underlying mechanism. During ferroptosis, mitochondrial cristae are reduced or disappear, and the mitochondrial membrane becomes ruptured and condensed [12]. Iron is normally present in the form of Fe^3+^ under physiological conditions, and its transport and storage are primarily regulated by TFR1, FTH1, and FPN1 [25,26]. TFR1 mediates the internalization of circulating Fe^3+^ into cells, where it is reduced to ferric ion (Fe^2+^), transported into intestinal epithelial cells, and released into the cytoplasmic labile iron pool (LIP) to exert physiological functions. In contrast, FPN is mainly responsible for exporting intracellular Fe^3+^. As the two key subunits of ferritin, FTL and FTH1 are regulated by IREB2, a critical transcription factor in iron metabolism. Inhibition of IREB2 significantly upregulates FTL and FTH1 expression, thereby initiating events associated with ferroptosis [27]. Sun et al. [28] demonstrated that HSPB1 acts a negative regulator of ferroptosis. In present study, we found that DON challenge significantly increased the Fe^2+^ content in liver, while FO supplementation reduced the Fe^2+^ concentration in DON-exposed pigs, which suggested that DON exposure disrupted iron metabolism, whereas FO supplementation attenuated the DON-induced imbalance of iron metabolism, thereby potentially inhibiting the initiation of ferroptosis. Moreover, our results on the mRNA expression of ferroptosis-related genes showed that DON downregulated the mRNA levels of HSPB1 and upregulated the mRNA levels of TF, FTH, SLC7A11, and TFR1, which indicate the activation of processes linked to ferroptosis. Liu et al. [29] also reported that dietary supplementation with 3 mg/kg DON increased the mRNA expression of FTL and FTH1, while decreasing the expression of FPN in the porcine duodenum. Cystine/glutamate antiporter system Xc-, composed of SLC7A11 and SLC3A2, is closely linked to cysteine metabolism [30,31,32]. This system exports glutamate and imports cystine, which is subsequently reduced to cysteine for the synthesis of GSH, a major intracellular antioxidant. GSH is oxidized under the action of GPX4, while ROS and lipid peroxides are reduced, thereby maintaining intracellular redox balance and mitigating oxidative damage [33,34]. In this study, DON contamination was found to upregulate the protein expression of FTH1, whereas FO supplementation downregulated their expression. DON exposure also downregulated the GPX4 activity and GPX4 protein expression, which are specific features associated with ferroptosis. However, FO attenuated the decrease in GPX4 content in liver. These findings were consistent with previous reports showing that DHA and EPA decreased SLC7A11 protein expression and enhanced cellular resistance to lipid peroxidation, thereby inhibiting process related to ferroptosis [35]. Since FTH1 and SLC7A11 were positively correlated with ferroptosis, their inhibition indicated that FO supplementation can inhibit processes related to ferroptosis in liver cells after DON challenge. These results together indicate that DON contamination promotes hepatic ferroptosis-related changes, and FO supplementation significantly alleviates these adverse alterations. To date, research on the role of FO in modulating ferroptosis remains limited. Our findings provide new insight into the protective role of FO against DON-induced liver injury, which may be partly mediated by inhibiting ferroptosis-related signaling.

Ferroptosis is a form of cell death characterized by lipid peroxidation. DON exposure elevated ROS levels, caused hepatic oxidative stress, and impaired the antioxidant system, ultimately leading to liver damage and cell death [23]. MDA, a primary end product of lipid peroxidation [36], has been shown to increase in serum following DON exposure, indicating disruption of the hepatic antioxidant balance and the onset of oxidative stress in piglets. In the present study, dietary supplementation with FO significantly reduced MDA concentrations in serum and liver. This finding was supported by Hassan et al. [37], who reported a substantial increase in MDA levels in serum and liver of chickens treated with DON. Further supporting the protective role of FO components, Yang et al. [38] demonstrated that FO counteracted hyperglycemia-induced elevation of MDA and reduction of GSH in human erythrocytes. Similarly, another study reported that EPA and DHA increased GSH and GPX4 levels while decreasing MDA content in the brains of pentylenetetrazole-exposed mice, suggesting an inhibitory effect on ferroptosis [39]. Another key biomarker of lipid peroxidation, 4-HNE, was also elevated upon DON exposure. However, FO supplementation significantly reduced 4-HNE levels. Similarly, Yang et al. [38] reported that DHA markedly decreased 4-HNE content in microglia cells following LPS challenge. Collectively, these results indicate that FO exerts protective effects on the antioxidant system and ferroptosis-related processes in piglets.

## 5. Conclusions

Dietary FO attenuated DON-induced liver injury in weaned piglets. The beneficial effects of FO may be the modulation closely associated with ferroptosis signaling pathways. Our findings provided a novel approach to prevent the harm caused by DON contamination.

## Data Availability

The original contributions presented in this study are included in the article/Supplementary Material. Further inquiries can be directed to the corresponding author.

## Supplementary Materials

The following supporting information can be downloaded at: https://www.mdpi.com/article/10.3390/ani16081234/s1, Table S1: Composition and nutrient contents of diets. Table S2: Dietary fatty acid composition of oils ^a^. Table S3: Primer sequences used for real-time PCR.

## Abbreviations

4-HNE	4-hydroxynonenal multidisciplinary
ACSL4	acyl-CoA synthetase long chain family member 4
AKP	alkaline phosphatase
ALOX15	arachidonate 15-lipoxygenase
ALT	alanine aminotransferase
AST	aspartate transaminase
DON	deoxynivalenol
FPN	ferroportin
FTH	ferritin heavy chain
FTL	ferritin light chain
GPX4	glutathione peroxidase 4
GSH	glutathione
HSPB1	heat shock protein beta-1
IREB2	iron-responsive element-binding protein 2
LPCAT3	lysophosphatidylcholine acyltransferase 3
SLC7A11	solute carrier family 7 member 11
System xc-	the cystine/glutamate transporter
TF	transferrin
TFR1	transferrin receptor 1
