# Supplementary material for: Fish Oil Ameliorates Deoxynivalenol-Induced Liver Injury Through Modulating Ferroptosis Signaling Pathway in Weaned Pigs
Source: Animals (Basel). 2026 Apr 17;16(8):1234. doi: 10.3390/ani16081234 (PMC13113518; doi:10.3390/ani16081234)
Supplement: Supplementary file 1 [file animals-16-01234-s001.zip › animals-4212632-supplementary.pdf]

Table S1: Composition and nutrient contents of diets.

Ingredients (%)	CO[1] diet	FO[1] diet
Corn	62.90	62.90
Soybean meal	7.50	7.50
Fermented soybean meal	8.00	8.00
Fish meal	5.00	5.00
Whey powder	5.00	5.00
Glucose	2.00	2.00
Corn oil	5.00	0.00
Fish oil	0.00	5.00
Calcium hydro phosphate	0.95	0.95
Limestone	0.80	0.80
Salt	0.30	0.30
L-Lysine hydrochloride (78%)	0.85	0.85
L-Methionine (98%)	0.29	0.29
L-Threonine (98%)	0.32	0.32
Tryptophan	0.09	0.09
Vitamin and mineral premix[a]	1.00	1.00
Nutrient composition [b]		
Digestible energy, MJ/kg	15.27	15.21
Crude protein (%)	17.47	17.47
Ca (%)	0.81	0.81
Total phosphorus (%)	0.62	0.62
Standard total tract digestible phosphorus (%)	0.36	0.36
Lysine (%)	1.52	1.52
SID- Lysine (%)	1.36	1.36
Methionine (%)	0.59	0.59
SID- Methionine (%)	0.55	0.55
Methionine + L-Cystine (%)	0.86	0.86
SID- Methionine + L-Cystine (%)	0.75	0.75
Threonine (%)	0.93	0.93
SID- Threonine (%)	0.80	0.80
Tryptophan (%)	0.26	0.26
SID-Tryptophan (%)	0.23	0.23

[1]CO: corn oil; FO: fish oil.

[a]Vitamin and mineral premix provided per kg of diet: Vitamin A, 5,521 IU; Vitamin D3, 2,200 IU; Vitamin E, 30 IU; Vitamin K3, 4 mg; Calcium D-Pantothenate, 20 mg; Nicotinic acid, 26 mg; Vitamin B12, 0.01 mg; Mn, 40 mg

($MnSO_4 \cdot H_2O$); Fe, 75 mg ($FeSO_4 \cdot H_2O$); Zn, 75 mg ($ZnSO_4 \cdot 7H_2O$); Cu, 100 mg ($CuSO_4 \cdot 5H_2O$); I, 0.3 mg (KI); Se, 0.3 mg (sodium selenite, Se-enriched yeast or Se-enriched Cardamine violifolia).

[b]The nutrients contents were analyzed values except ATTD phosphorus, standard ileum digestible lysine, methionine, methionine + cystine, threonine and tryptophan which were calculated values.

Table S2: Table 2 Dietary fatty acid composition of oils.[a]

Fatty acid	CO[1]	FO[1]
	% total fatty acids	
14 : 0	0.4	5.3
16 : 0	15.0	20.3
16: 1 (n-7)	0.5	6.0
18 : 0	2.4	3.7
cis 18: 1 (n-9)	27.9	17.4
cis 18: 2 (n-6)	47.4	20.3
18 : 3 (n-3)	1.3	1.4
20: 4 (n-6)	0.1	0.8
20: 5 (n-3)	ND[b]	11.6
22: 6 (n-3)	0.9	7.9
Total (n-6) PUFA[1c]	47.6	21.5
Total (n-3) PUFA[c]	2.2	20.9
(n-6) : (n-3)[c]	21.8	1.0

[1]CO: corn oil; FO: fish oil; PUFA: polyunsaturated fatty acids.

[a]The most abundant fatty acids are listed. The detection limit for each fatty acid was 0.001 mg/g. [b]ND, Not detected.

[c]Total (n-6) PUFAs and total (n-3) PUFAs are the sum of all (n-6) or (n-3) PUFAs detected.

Table S3: Primer sequences used for real-time PCR.

Genes	Forward (5′-3′）	Reverse (5′-3′)
β-actin	TGCGGGACATCAAGGAGAAG	AGTTGAAGGTGGTCTCGTGG
TFR1	CGAAGTGGCTGGTCATCT	TGTCTCTTGTCTCTACATTCCT
HSPB1	CTCGGAGATCCAGCAGACT	TCGTGCTTGCCCGTGAT
SLC7A11	GCCTTGTCCTATGCTGAGTTG	GTTCCAGAATGTAGCGTCCAA
FTH	ACTTCATTGAGACGCATTAC	GATGTTCTGGTAGGACTTATAG
FPN	GGTTCTTACTTCCTGCTATATC	ACTGGTCAATCCTTCGTATT
TF	TGAGCTTGGATGGAGGCTAC	CCACAGCCAAATACCCTTTCT
ACSL4	CTCCCTTCCGCCAGCTTC	CGTGCTTTCAATCAGAGGCG
ALOX15	TCCACTGGGTCGTCGTTCTA	ACTGAATTCCGTCTCCTTGCC
